# Cell matrix adhesions in cancer: The proteins that form the glue

**DOI:** 10.18632/oncotarget.17265

**Published:** 2017-04-20

**Authors:** Mazvita Maziveyi, Suresh K. Alahari

**Affiliations:** ^1^ Department of Biochemistry and Molecular Biology, Stanley S. Scott Cancer Center, Louisiana State University Health Sciences Center, New Orleans, LA, USA

**Keywords:** integrins, cancer, focal adhesions

## Abstract

The main purposes of Integrin-mediated cell contacts are to interpret bi-directional signals between the extracellular environment and intracellular proteins, as well as, anchor the cell to a matrix. Many cell adhesion molecules have been discovered with a wide spectrum of responsibilities, including recruiting, activating, elongating, and maintaining. This review will perlustrate some of the key incidences that precede focal adhesion formation. Tyrosine phosphorylation is a key signaling initiation event that leads to the recruitment of multiple proteins to focal adhesion sites. Recruitment and concentration of proteins such as Paxillin and Vinculin to Integrin clutches is necessary for focal adhesion development. The assembled networks are responsible for transmitting signals back and forth from the extracellular matrix (ECM) to Actin and its binding proteins. Cancer cells exhibit highly altered focal adhesion dynamics. This review will highlight some key discoveries in cancer cell adhesion, as well as, identify current gaps in knowledge.

## INTRODUCTION

During metastasis, cancerous cells have to migrate through the extracellular matrix (ECM) for intravasation into blood and lymphatic vessels. The early steps of metastasis involve the coordination of intracellular signals and the microenvironment to allow detachment of the cell from the primary site, and this is first demonstrated by a change in the cells shape. The cells become densely packed and have a reduced amount of protrusions. For stability, cells are linked to the ECM through cell adhesion molecules. In cancer, many cell adhesion molecules participate in this cell-matrix linkage [1].

Focal adhesions (FAs) are large protein complexes that connect the cell cytoskeleton to the ECM through integrins. Integrins are cell adhesion proteins with α and β transmembrane heterodimers that play a major role in converging signals from the cell membrane to the inside of the cell [2]. The cytosolic portions of both transmembrane heterodimers interact with a number of cytoskeletal proteins and signaling molecules. These heterodimers act as receptors for ECM proteins including Collagen, Laminin, Thrombospondins, Vitronectin and Fibronectin [3, 4]. The two major roles of integrins are to convey outside-in and inside-out signaling. For inside-out signaling, proteins containing FERM domains, such as Talins and Kindlins, bind to Integrin cytoplasmic tails to trigger conformational changes [5]. Many FA proteins contain these FERM domains which are discussed later on in this review. The conformational changes triggered by these FERM domains lead to integrin activation. In mature FAs, Integrins are active, erect, and bound to their ligand [6].

For outside-in signaling, the binding of the active Integrin to their respective immobilized ligand results in the recruitment of more adaptor proteins which results in a strengthened cytoskeleton at the FA [7]. Whilst bound to the ligands, Integrins act as force sensors and force transmitters [8, 9]. The ECM can exert forces on Integrins that transfer to the Integrin-associated FA proteins and expose new binding sites for further intramolecular interactions [9]. Furthermore, cells also respond to this altered tension by inducing changes in gene transcription [10]. Oppositely, the cytoskeleton of the FA generates forces that allow the Integrins to pull ECM proteins [11].

The ability of Integrins to connect to the cytoskeleton and transmit forces speaks to its critical role in FA turnover. Because of this, tumor cell migration and invasion rely on Integrin activation/deactivation, as well as, their crosstalk with oncogenes. Integrin signaling is a well orchestrated but complicated phenomenon. Understanding the protein networks that regulate Integrin activation will give us a better understanding of how cells regulate their adhesion.

Cell-matrix adhesion sites were first observed more than 40 years ago in chick heart fibroblasts by electron microscopy and interference reflexion microscopy [12–14]. These contacts were first identified to be 2-10 μm long, and 0.25-0.5 μm wide [14]. Since then, studies have found that FA size ranges from 0.25 to 10 μm, depending on cell mobility [15]. For example, rapidly moving cells such as *Dictyostelium discoidem* cells display small FAs [16], while cells with prominent FAs move slower [17, 18].

Intracellularly, FA proteins participate in a number of cell signaling pathways. Tyrosine phosphorylation is a key signaling event that leads to the recruitment of multiple proteins to FAs [19]. Although the kinases ILK, PAK, Abl, Csk, PYK2 and PKC are present in FAs [12], Focal Adhesion Kinase (FAK) and Src are the most dominant kinases in FA signaling [20]. Tyrosine phosphorylation by these kinases leads to a recruitment of cytoskeletal proteins, such as Paxillin [21], small GTPases, tyrosine phosphatases and other enzymes.

Cancer cells participate in different types of directed cell migration including: chemotaxis (chemoattractant gradient), haptotaxis (environmental gradient), electrotaxis (electrical attractant), and durotaxis (rigidity attractant) [22]. Environmental sensing by the cancer cells allows them to interpret various signals that are mediated by FAs. ECM generated stiffness induces and cooperates with cell-matrix forces applied by cells [23]. Cells are capable of sensing many characteristics of the ECM such as rigidity and anisotropy [7]. In fact, ECM composition is a key contributor of patient survival [24]. It has been shown that Collagen fibers straighten as the breast tumor progresses [25]. Detection of ECM rigidity by Integrins through Myosin II leads to downstream signaling cascades through FAs [26]. For example, the FAK/Paxillin/Vinculin signaling pathway transmits force information to allow the cell to migrate in different ECM rigidities [27]. In fact, a study showed that Null-FAK cells are unable to detect changes in ECM rigidity [28].

Most FA proteins contain multiple binding sites for other proteins; therefore supramolecular structures tend to form within these sites. The regulation of these protein-protein interactions plays a key role in the development and turnover of adhesion sites. Since it has been shown that the size and composition of the FA contributes to cellular functions such as migration and mechanosensing [29], it is important to study the different components of FA complexes. This review will describe some of the key proteins involved in FA signaling.

## INITIATION OF FA ASSEMBLY

Mature FAs are dependent on Actin filament assembly at the leading edge, also referred to as Actin flow [30]. In order for this to happen, a number of proteins have to be recruited to the site. Tyrosine phosphorylation is a key signaling event that leads to the recruitment of multiple proteins to FAs [19]. Although not as common, serine phosphorylation also plays an important role in adhesion formation. Focal Adhesion Kinase (FAK) and Src are the most dominant kinases in FA signaling [20]. Other kinases have been shown to participate in this labyrinthine signaling pathway, but inactivation of FAK and Src averts FA assembly. Therefore, it is important to understand the importance of FAK in FA assembly.

### *FAK* and *Src*

FAK, a non-receptor tyrosine kinase, is the central regulator of Integrin-mediated FA assembly. FAK was first found to be activated by the ECM or growth factors and its tyrosine phosphorylation is associated with the formation and turnover of FAs [31]. In fact, Null-FAK fibroblasts exhibit an abundance of FAs [32]. Immunohistochemical analysis revealed increased FAK expression in highly malignant human breast and colon cancers [33]. Since FAK is both a signaling kinase and an adaptor/scaffold protein, it is involved in various signaling pathways including those that contribute to cell migration and angiogenesis [34, 35].

In the past ten years, the molecular mechanisms of FAK regulation has been uncovered in detail. The N-terminus of FAK contains a FERM (Four-point-one, Ezrin, Radixin, Moesin) domain that is important for protein:phosphoinositide and protein:protein interactions [36, 37]. The C-terminus contains a four-helix bundle FAT (Focal Adhesion Targeting) domain that is critical for FAKs targeting to FAs through the protein Paxillin [38]. These FERM and FAT domains are the dominant protein:protein binding domains of FAK [39]. For example, Met interacts with the FERM domain to promote hepatocyte growth factor-mediated cell invasion [40]. The FERM domain also interacts with the FAK kinase domain to block the active site from accessing ATP and other substrate binding sites for activation [38, 41]. Mutation of the FERM domain at K38 interrupts the FERM:kinase domain interaction and leads to FAK activation [42]. In summary, the self-inactivation of FAK consists of the FERM domain directly interacting with the kinase domain.

Posttranslational modifications are the major regulatory activators for FAK, mainly phosphorylation. The major autophosphorylation site during FA signaling is Tyr397 [34, 43]. Phosphorylation at this site establishes a motif that is recognized by proteins containing SH2 domains, including Src kinase [44]. Many proteins have been identified that contribute to FAK phosphorylation. Multiple factors, including increased p130RhoGEF expression, contribute to the localization of FAK to the elicited adhesion site [45]. In fact, a mutation in the pleckstrin homology (PH) domain of p130RhoGEF inhibits FA assembly [45]. As stated earlier, FAK may be activated by growth factors. Of those growth factors, platelet-derived growth factor (PDGF) treatment of cells increases autophosphorylation of FAK at Tyr397 [46]. Also, PDGF stimulation of the cells induced Akt-mediated phosphorylation of FAK at Ser695 and Thr700 to enhance Integrin signaling [46]. In the early stages of adhesion development, FAK responds to increased Integrin clustering due to ECM rigidity by autophosphoylation at tyrosine 397 and subsequent activation [47–50]. This autophosphorylation site creates a motif that allows recognition of proteins with SH2 domains, such as the p85 subunit of PI3K, Phospholipase Cγ (PLCγ) and the SRC-Family Kinases (SFKs) [34]. Even though this autophosphorylation is an indispensable event for FA signaling, FAK is phosphorylated at other sites as well.

The oncogene Migration and invasion enhancer 1 (MIEN1) regulates cell migration by inducing phosphorylation of FAK at Tyr-925 [51]. *MIEN1* is located in the chromosomal region 17q12-21 and is typically amplified along with its neighboring gene *ERBB2* in breast cancer [52]. Its many functions include regulating Actin cytoskeletal dynamics and influencing expression of ECM-degrading proteases and angiogenic factors [51, 53]. Down regulation of MIEN1 leads to a reduction in FAK phosphorylation at Tyr-925, as well as reduced phosphorylation of Akt, Erk1/2 and NF-κB [51]. Analysis of FAK activation is of paramount importance to any FA signaling study. Many FAK-activating proteins have been characterized, including p130RhoGEF [45], PDGF [46] and MIEN1 [51], however, the complexity of this signaling pathway suggests that multiple other factors are waiting to be discovered.

FAK autophosphorylation also leads to the activation of the tyrosine kinase Src [54]. As discussed earlier, PDGF is a growth factor that activates FAK. Epidermal growth factor (EGF) is another growth factor that activates FAK, as well as Src [55]. Src tyrosine kinase works alongside with active FAK to facilitate the phosphorylation of many FAK-associated proteins. Src-family kinases are essential for the formation and maturation of FAs and are highly activated in many human cancers [56, 57]. Genomic amplifications and activating mutations of Src are rare in human cancers so this hyperactivation is often induced by upstream phosphatases or kinases [57]. Src has several functional domains, including, a 14-carbon myristic acid moiety attached to a SH4 domain, a SH3 domain followed by an SH2 domain, an SH2 linker, kinase domain and C-terminal regulatory domain. Src is capable of interacting with the plasma membrane through its 14-carbon N-terminal fatty acid moiety [57, 58]. After activation, its SH3 domain interacts with Actin filaments to transmit signals from adhesion receptors to cytoskeletal proteins [59].

Though FAK autophosphorylation engenders Src activation, concurrent signaling events may occur. Although the precise order in which each activating event occurs is unknown, FAK and Src do work in concert to activate other proteins. For example, in response to extracellular Fibronectin, mechanical stretching of the substrate domain of Cas exposes Src-specific phosphorylation sites [60], and in order for this Src-mediated phosphorylation to occur, Cas must be bound to FAK [61]. Src activates various downstream signaling pathways through its interactions with FAK, epidermal growth factor receptors (EGFRs), hepatocyte growth factor receptor (C-Met or HGFR), Integrin cell adhesion receptors, G protein-coupled receptors, and other cytoskeleton components [62, 63].

Many protein:protein complexes are found at FA sites. It has been shown that recruitment of FA proteins such as Vinculin and Paxillin precedes tyrosine phosphorylation [19]. This justifies the importance of the localization of the tyrosine kinases FAK and Src to these nascent adhesion sites. These initial phosphorylation events lead to the activation of other kinases and phosphatases while creating a substrate for SH2-containing proteins. Although the kinases ILK, PAK, Abl, Csk, PYK2 and PKC are present [12], Focal Adhesion Kinase (FAK) and Src are the most dominant kinases in FA signaling [20]. The FAK-Src symbiotic relationship leads to the subsequent recruitment of many proteins to FA sites. FAK and Src bind different partners to uniquely regulate FA dynamics. Some studies only choose to assess FAK activation as a determinant of FA function. Other kinases such as ILK, PAK, Abl, Csk, PYK2 and PKC may be excluded in adhesion studies but it is imperative that Src kinase function be gauged, along with FAK.

## PROTEIN RECRUITMENT

Immature FAs are detected under the microscope as “Actin dots” that are rich in Integrins, Paxillin and Vinculin [64]. Recruitment and concentration of Paxillin and Vinculin to Integrin clutches is necessary for FA development. Though both proteins have unique roles in adhesion signaling, they play a fundamental role in recruiting key proteins to the adhesion interactome. Their ultimate goals are to transmit signals back and forth from the ECM to Actin and its binding proteins. Formation of Actin filament stress fibers precedes FA formation [65].

### Paxillin

**Figure 1:**
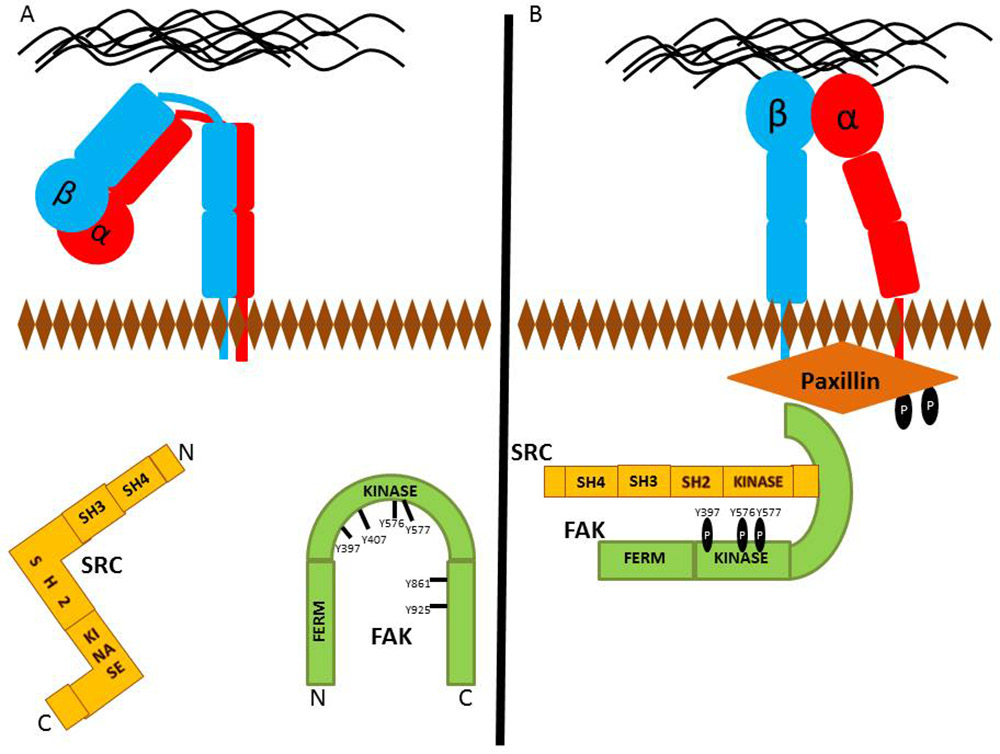
Protein recruitment to future FA site **(A)** Detached FAs typically have inactivated Integrins, as well as inactive FAK and Src kinases. **(B)** FAK phosphorylation at Y397 leads to the activation of Src. In the early events of FA formation, localization of Src and FAK to FA sites results in Paxillin phosphorylation at tyrosines 118 and 31.

Activation of the FA scaffold protein, Paxillin, occurs subsequently after kinase recruitment [66]. The active FAK-Src proteins phosphorylate Paxillin, and various other adhesion molecules [67]. Localization of active Src and FAK to FAs results in the phosphorylation of Paxillin at tyrosines 31 and 118 as shown in Figure 1 [68]. Paxillin is an adaptor/scaffolding protein that participates in a number of cell signaling pathways. The C-terminus contains two LIM (Lin11, Isl-1, Mec-3) domains that are necessary for targeting to FAs [69, 70]. These LIM domains are also important for binding to FA regulatory and structural proteins such as Tubulin and Protein Tyrosine Phosphatase Non-Receptor Type 12 (PTPN12) [71, 72].

**Figure 2:**
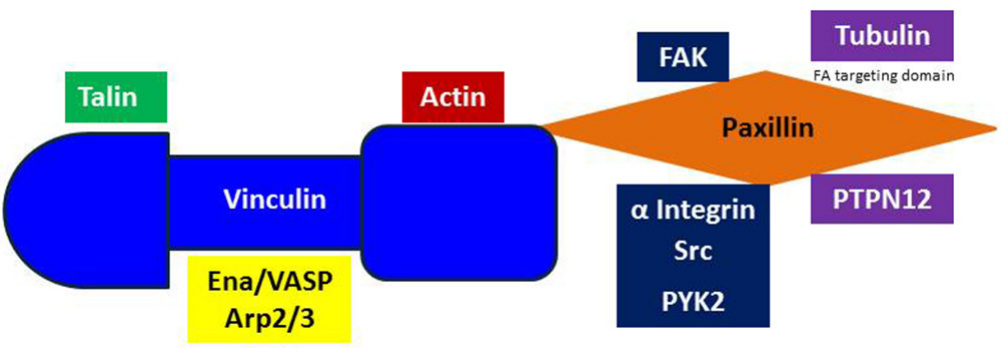
Vinculin and Paxillin binding Vinculin is a direct binding partner of Paxillin, Talin, Arp2/3, Actin and the Ena/VASP proteins. Paxillin binds to PYK2, Integrin, Src, Tubulin and PTPN12. The LIM2 and LIM3 domains of Paxillin are necessary for FA targeting.

The N-terminus of Paxillin contains leucine and aspartate rich motifs that are critical for its interaction with FAK, Vinculin, PYK2 and a number of other FA proteins (Figure 2) [73]. The proline-rich region of Paxillin also binds to Src [74]. Furthermore, Paxillin contains many serine and threonine phosphorylation sites that are phosphorylated by a number of stimulants, including Receptor for Activated C Kinase 1 (RACK1), a Src kinase inhibitor, and the serine/threonine-protein kinase, p21-activated Kinase (PAK) [74]. In the early stages of FA formation, Paxillin experiences phosphorylation of tyrosine 118, and serines 85, 188 and 190 [75, 76]. Paxillin is one of the first proteins present in nascent adhesions and recruits many structural and regulatory proteins to the cell adhesion site [74, 77]. Since Paxillin has many direct binding partners, understanding the orchestration of signaling events will help to fathom the specificity Paxillin holds for each dynamic process. This makes Paxillin an attractive target for anti-metastatic drugs since its inhibition prevents the formation of FAs.

### Vinculin

Vinculin is an F-Actin-binding protein that also appears in the early stages of adhesion formation [78]. Paxillin, F-actin and Talin are necessary for Vinculin recruitment to FA sites [78–82]. Vinculin is a direct binding partner of Paxillin and its interaction is important for coordinating force-induced signals (Figure 2) [27, 83]. Vinculin deficient cells are less stiff than normal cells and have lower traction forces due to a reduction in Vinculin's tension sensing capabilities [84, 85]. Vinculin is a tension sensor that is recruited to the FA site in response to mechanical forces by conveying signals from the ECM to Actin filaments [86].

In addition to its role in mechanosensing, Vinculin directly interacts with a number of proteins that are important for Actin cytoskeletal dynamics. The α-helical Vinculin tail directly binds to Actin (Figure 2) [81, 87]. Immediately after binding, Vinculin dimerizes and begins to assemble bundles of Actin filaments [87, 88]. The polymerization of these filaments occurs by Vinculin-mediated recruitment of G-Actin to form a nucleus for polymerization [81]. Vinculin then recruits and directly binds to the Arp2/3 Actin polymerization complex [89]. Also, Vinculin directly interacts with molecules such as the membrane phospholipid, PIP2, and the cytoskeletal adaptor protein, Vinexin to increase FA size during the elongation phase of FA assembly [90, 91]. These interactions are necessary for Actin polymerization to occur.

Vinculin and Talin interactions are necessary for strong Integrin-cytoskeleton bonds. Vinculin knockdown in cells increases cell migration due to increased Paxillin and FAK phosphorylation, as well as, an increase in FA turnover [92, 93]. Vinculin has recently been discovered in prostate cancer exosomes [94]. Though there is a gap in knowledge about why Vinculin is present in exosomes, these secreted micro vesicles may be isolated from the serum of patients and identification of Vinculin could serve as another prognostic marker.

Vinculin is an indirect target of the common arthritis drug, Celecoxib. Celecoxib is a nonsteroidal anti-inflammatory drug that inhibits cyclooxygenase-2 (COX-2) [95]. This inhibitor was first published as a chemopreventative agent in colon carcinogenesis in 1998, just shortly after its synthesis [96]. Since then, the tumor preventative effects of Celecoxib have been seen in many other cancer models, including urinary bladder cancer [97], prostate cancers [98, 99], and breast cancer [100]. Recently, Celecoxib has been shown to exert anti-gastric cancer effects by down regulating the expression of 8 genes, including Vinculin [101]. The other 7 genes, Cysteine and glycine-rich protein 1 (*CRP1*) [102], Thrombospondin 1 (*THBS1*) [103], Myosin light chain 9 (*MYL9*) [104], Filamin A (*FLNA*) [105], Actinin alpha 1 (*ACTN1*) [106], Laminin subunit gamma 2 (*LAMC2*) [107] and Claudin 1 (*CLDN1*) [108] are also linked to cell adhesion. These research findings suggest that Celecoxib exerts its anti-cancer functions by down regulating the expression of cell adhesion molecules. This further stresses the importance of studying FAs and the therapeutic potential that could be obtained from those studies.

## ACTIN ASSEMBLY

**Figure 3:**
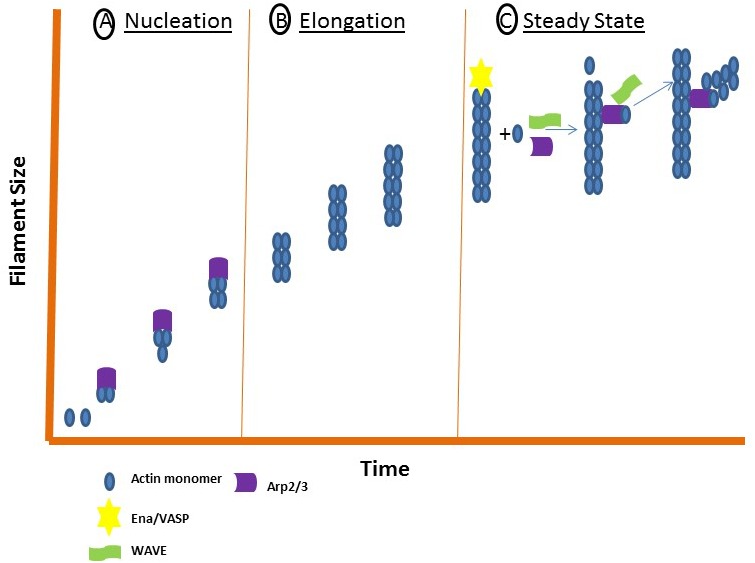
The three stages of filament assembly Actin filament assembly requires three stages: a nucleation, elongation and steady state phase. **(A)** During nucleation, the Actin monomers are converted to stable multimers through nucleating proteins such as Arp2/3. **(B)** During elongation, monomers are rapidly added to each end. **(C)** During the steady state phase, WAVE and Ena/VASP interact to enhance Arp2/3 filament assembly.

Actin is one of the most abundant proteins in eukaryotic cells. Polymerization of Actin into microfilaments is required for a sturdy cell-matrix adhesion point. This assembly requires three phases: a nucleation, elongation and steady state phase. During nucleation, Actin monomers are converted to stable multimers. These multimers are then rapidly elongated by adding monomers to both ends. Finally, the steady state phase is achieved once equilibrium is reached between the polymerization at one end and the disassembly at the opposite as shown in Figure 3. This three-step process requires many proteins but we will focus on the three most studied assembly pathways that include the Arp2/3 complex, the Ena/VASP family, Formins and Talin.

### Arp2/3 complex

Actin nucleation is the mechanism by which Actin monomers form into a stable multimer. Actin Related Protein 2 (Arp2) and Actin Related Protein 3 (Arp3) were the first Actin nucleators to be discovered [109]. These two proteins form a complex known as the Arp2/3 complex. For activation, this complex relies on nucleation promoting factors such as Wiskott-Aldrich Syndrome Protein (WASP) and WASP-family verprolin-homologous protein (WAVE) [110, 111]. Cortactin has also been shown to cooperate with N-WASP to activate the Arp2/3 complex [112, 113]. The precise mechanism that the Arp2/3 complex uses for Actin nucleation is heavily debated [109–111]. For example, one study showed that the Actin binding protein Caldesmon increases Arp2/3-mediated branching activity by promoting the binding of newly polymerized Actin to the Arp2/3 complex [114]. Another study showed that accelerated release of WASP proteins from the Actin-Arp2/3 complex accelerated branch forming [115], suggesting that WASP dissociates from the Actin-Arp2/3 complex prior to Actin filament growth.

Cells with *HER2* gene amplification have increased co-expression of both WAVE2 and Arp2/3 [116]. This is of great significance because *HER2* gene amplification is a poor prognostic indicator in early breast cancer. Furthermore, HER2 status plays a role in selecting the most effective treatment option for both early and advanced breast cancer. The reason why there is co-amplification of these genes is unclear, especially since the genes are all located on different chromosomes. Arp2/3 mRNA and protein expression are often upregulated in some other invasive cancer cells as well, including colorectal and lung adenocarcinoma's [117–119]. Though these other invasive cancers are not associated with frequent *HER2* amplification, its co-amplification patterns in breast cancer need to be further explored.

Two highly effective small molecule inhibitors, CK-666 and CK-869 bind the Arp 2/3 complex and reversibly inhibit Actin nucleation [120]. Both molecules have distinct binding sites with CK-666 blocking the movement of the complex proteins to their activated conformation [121]. CK-666 treatment of the human ovarian carcinoma cell line, A2780, resulted in a collapse of lamellipodia [122], while treatment of fibroblasts with CK-869 caused blebbing [123]. CK-636 is a similar inhibitor that has a thiopene rather than the fluorobenzene ring of CK-666 but has a much lower IC_50_ [120]. CK-869 alternatively binds to an off-hand cavity on Arp 3 that destabilizes its interaction with Arp 2 [121]. Its analog, CK-548, has *ortho*-hydroxy and *meta*-chlorine substituent's where CK-869 has methoxy groups in the *ortho* and *para* positions [120]. CK-869 is acknowledged as the more effective inhibitor because a study showed that CK-548 does not effectively inhibit comet tail formation by *Listeria* [120]. CK-869 inhibition in the MCF10A mammary gland cell line disrupted lamellipodia formation and cell spreading [124].

The ability of Arp complex inhibitors to prevent cell adhesion speaks to the importance of adhesion assembly. Although the disruption of lamellipodia upon treatment with Actin nucleation inhibitors is universal, several studies have found no disruption of monolayer integrity or cytotoxicity of normal epithelial cells [124, 125]. Given that treatment of cells with these compounds prevents Actin branched networks, these inhibitors could serve as effective cancer therapeutics. Recruitment of key FA proteins such as FAK and Paxillin to FA sites is important but if assembly of the Actin filament is perturbed, FAs will not form.

### Ena/VASP protein family

The nucleation-promoting factor and Arp2/3 activator, WAVE, directly binds to Ena/VASP to enhance Arp2/3 filament assembly (Figure 3) [126]. The Ena/VASP protein family regulates Actin assembly and has been implicated in fibroblast migration, T cell polarization and axon guidance [127]. These proteins are targeted to areas of Actin remodeling, such as the leading edges of invadopodia, lamellipodia, filopodia and Actin-dependent cell-cell contacts [127–129]. Actin proteins are non-symmetrical structures with two ends, each referred to as a “barbed end” and “pointed end” [130]. Polarized growth of the filament is regulated by proteins that cap their ends to block the assembly of polarized filaments [130]. Disassembly of the filaments occurs at the pointed end, while elongation occurs at the barbed end [130]. Ena/VASP regulatory molecules protect Actin filaments from capping proteins at their barbed ends [131, 132]. Also, Ena/VASP proteins increase the density of Arp2/3-dependent Actin filament branches during Actin polymerization [127].

Proteins from this family regulate many cell motility pathways and are associated with poor clinical outcome in breast cancer patients [133, 134]. Ena/VASP proteins are uniquely linked to Profilin-1 (Pfn1), a G-Actin binding protein that is down regulated in breast, hepatic and pancreatic adenocarcinomas [135–137]. In breast cancer, down regulation of Pfn1 in MDA-MB-231 cells leads to a highly motile phenotype that was orchestrated by Ena/VASP proteins [138]. In fact, silencing of Pfn1 in these cells enhanced the localization of VASP to their leading edge to enhance cell motility [138]. The targeting of Ena/VASP proteins to the leading edge and subsequent enhanced motility hints to its importance for adhesion dynamics in cancer cells.

### Formins

Like Ena/VASP proteins, the Formin protein family is responsible for the formation of unbranched Actin filaments. The Formin family consists of fifteen genes that play a role in many cellular processes, including cell adhesion, migration, stress fiber formation, and development [139]. Formins are defined by their highly conserved C-terminal Formin homology 2 (FH2) domain, which nucleates Actin monomers [140]. The N-terminus contains a FH1 domain that recruits Profilin-Actin monomers to grow the filament [141].

FHOD1 is a Formin that is necessary for cell spreading, adhesion maturation, and the transduction of adhesive force [142]. It is the major Formin transcriptionally upregulated during the epithelial to mesenchymal transition (EMT) of oral squamous carcinoma cells (OSCC) [143]. EMT is a process by which non-motile epithelial cells gain an invasive, mesenchymal phenotype [144]. Knockdown of FHOD1 results in the repression of elongation, migration and invasion of SCC-43B OSCCs [143]. As a cell undergoes the EMT, it experiences an increase in Actin organization, motility and invasiveness mainly due to an increase in the expression of mesenchymal markers. The up-regulation of FHOD1 during this process suggests that it is a mesenchymal marker. This new fibroblast-like morphology has to have dynamic Actin assembly/disassembly capabilities. Arp2/3, Ena/VASP and Formins are crucial for Actin nucleation but require proteins like Talin to enhance the length of the FA.

### Talin

Talin is a critical component of cell contacts and the primary determinant of FA length [145]. Talin binds to many adhesion proteins, including Actin, FAK, Vinculin, and Integrins to enhance the strength of FAs [146–149]. Talin acts as a linker between Actin and Integrins to allow the transmission of biochemical signals and force across the cell membrane [150, 151]. Specifically, the interaction between Talin and β-Integrin triggers a conformational change in the Integrin extracellular domain that promotes a stronger affinity to the ECM [152]. Talin binding to β Integrins through its FERM domain is what induces this conformational change in Integrins that promotes its affinity for the ECM [153, 154].

Not only is Talin's role as a linker important for strong adhesions, but its role as an Integrin activator is significant as well [155]. In fact, downregulation of Talin leads to aberrant folding and processing of Integrins [156]. Talin overexpression is generally correlated with poor prognosis and this overexpression has been noted in triple negative breast cancers, nasopharyngeal carcinoma, and human hepatocellular carcinomas [157–159]. Interestingly, Talin 1 has been shown to have an opposite effect in prostate cancer because it impairs cell adhesion and motility [160]. Actin assembly is important for anchoring the cell, but the turnover of the FAs is essential to cell migration and invasion.

Actin filament disassembly is required for cell spreading and migration of cells. A key indicator of FA turnover is proteolysis of Integrins, FAK and Talin [161–163]. RhoGTPases promote FA turnover through FAK-mediated suppression [164]. Although much is known about Actin assembly in cancer, the opposite is evident for Actin disassembly. However, it is known that filaments grow until they are capped by proteins, such as those from the Ena/VASP family [165]. Extracellular cues, including substrate tension information instructs Actin assembly machinery on how long the particular FA should be. In the absence of such external signals, it is unknown which intrinsic signals regulate Actin assembly.

## THE CANCER CELLULAR ADHESOME

In recent years, novel proteins have been linked to FA regulation in cancer cells. Using proteomic analysis, a 2,413-protein Integrin adhesion complex has been identified [166]. These proteins are grouped into four axes of core adhesion machinery comprising the FAK-Paxillin, Talin-Vinculin, α-Actinin-Zyxin-VASP, and ILK-PINCH-Kindlin pathways [166]. The FAK-Paxillin, Talin-Vinculin, and α-Actinin-Zyxin-VASP have been well studied in cancer cells but not all constituents in the pathways have been discovered. Alterations in gene expression of these FA molecules can either lead to a change in the size of each cell-matrix contact, or cell death.

Targeting of FA proteins has been shown to sensitize cancer cells to treatment [167]. In MCF7 breast cancer cells, knockdown of FAK, Integrin linked kinase (ILK), Talin, and Zyxin leads to enlarged FAs [168]. Furthermore, Protein Phosphatase 1 Regulatory Subunit 12B (PPP1R12B), Homeodomain Interacting Protein Kinase 3 (HIPK3), Rac2, and Tropomyosin 1 (TPM1) inhibit Insulin-like Growth Factor 1 Receptor (IGF1R) driven migration of MCF7 cells [168]. These proteins enlarge FAs to anchor the cell to the matrix and reduce the rate of disassembly.

Inhibition of the serine/threonine kinase, NEK3 also leads to enlarged FAs in MCF7 breast cancer cells. The NimA related kinase (NEK) family is known for controlling cell cycle progression in response to genotoxic stress. NEK3, in particular, regulates Prolactin-mediated cytoskeletal reorganization in breast cancer cells [169]. Prolactin signaling also activates NEK3 at threonine 165 [170]. Inhibition of the NEK3 Thr-165 phosphorylation results in actin cytoskeleton reorganization and increased FA size [170]. Overexpression of NEK3-T165V in cancer cells led to a decrease in migration [170]. The coordination of adhesion assembly and disassembly is delicately orchestrated. The inhibition of MCF7 cell migration seems to be the consequence of enlarged FAs. This hints to an increase in the rate of Actin assembly and a decrease in the rate of Actin disassembly. Enlarged FAs are not always a consequence of the inhibition of cancer cell migration; some proteins can inhibit cell migration without enlarging the FAs.

Transformation-related protein 63 (p63) is a member of the p63/p53/p73 family of transcription factors that also induces cell cycle arrest and apoptosis [171]. This family of transcription factors contains three highly conserved domains: a transactivating domain, a DNA binding domain, and an oligomerization domain [171]. Alternative splicing of p63 brings forth two different isoforms: those with or without the full length transactivating domain [172]. Expression of the truncated isoform (ΔNp63) in basal-A triple-negative breast cancer (TNBC) cell lines resulted in a reduction of cell adhesion, which led to cell death [173]. Opposite effects have been demonstrated in squamous cell carcinomas of the esophagus (ESCC). Another group suggested that the amplification of *TP63* that leads to the hyper-expression of ΔNp63 seen in ESCC is what promotes cell adhesion [174]. In fact, inhibition of p63 in the TE13 squamous esophageal carcinoma cell line altered expression of cell adhesion proteins [174]. P63 has both oncogenic and suppressive capabilities that all eventually lead to a regulation of cell adhesion dynamics. The ΔNp63 isoform in particular seems to control these effects since the full-length protein has not been shown to affect cell adhesion. Though ΔNp63 alters the expression of some of the key FA proteins, the exact mechanism by which it exerts its functions is unknown.

Approximately 15-20% of newly described breast cancer cases are triple-negative breast cancers (TNBCs). This subtype is highly invasive and has the greater probability to metastasize. A few pharmacological inhibitors have been developed for reducing TNBC cell adhesion. AMPI-109 selectively targets TNBC cells by activating pro-apoptotic caspases-3 and 7, DNA fragmentation, and PARP cleavage [175]. Studies performed in BT-20 TNBC cell lines showed that AMPI-109 treatment impairs cell migration and invasion by reducing levels of the oncogenic tyrosine phosphatase, PRL-3 [175]. Further investigations of this showed that knockdown of PRL-3 or AMPI-109 treatment deactivate the Src and ERK signaling pathways, which reduces RhoA and Rac1/2/3 GTPase protein expression [176]. On the contrary, PRL-3 over expression increased adherence to and degradation of TNBC cells to the ECM component Laminin [176]. *In vivo* animal models further demonstrate that PRL-3 inhibition reduces growth of TNBC tumors [177]. These studies were the first to link PRL-3 to the FA pathway in TNBC cells. The fact that the effectiveness of the AMPI-109 is in its ability to disrupt cell adhesion dynamics speaks to the importance of fully understanding all of the components of the FA-signaling pathway.

## CLOSING REMARKS

**Figure 4:**
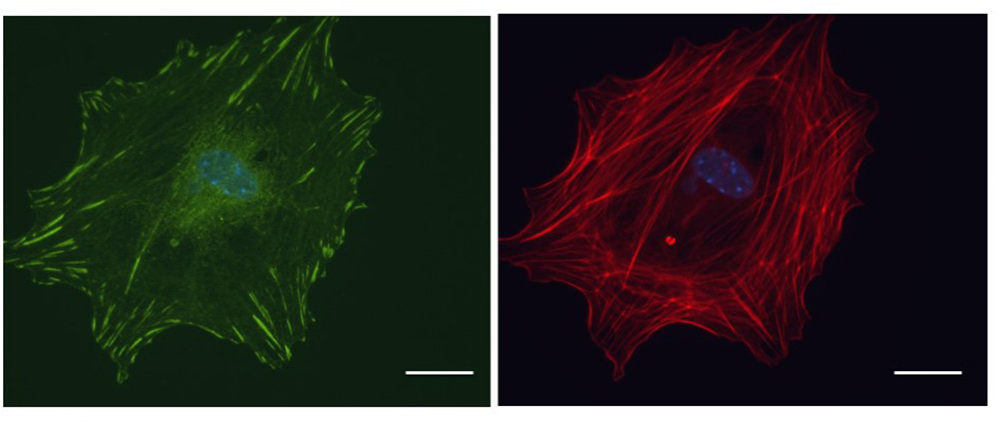
Microscopic visualization of FAs Primary tumor cells isolated from MMTV-PyMT tumors were plated on a Fibronectin-coated cover slip and labeled with Vinculin (green) to visualize FAs and DAPI (blue) to visualize the nucleus. Phalloidin (red) staining is shown in the right panel. Scale bar: 10μm.

Adhesion of a cell to the ECM regulates cancer cell morphology, migration, survival, and proliferation. The proteins involved in FA assembly serve as markers for standard Western Blot or Immunofluorescence techniques using lysed or fixed cells (Figure 4). However, live cell imaging allows us to study FA strength, turnover, and polymerization in real-time. The dynamics of the cytoskeleton near the edge of the cell can be visualized by using total internal reflection fluorescence (TIRF) microscopy. To use this, FA proteins are genetically tagged to fluorescent proteins then introduced into cells. Once the imaging has started, only the fluorophores within 100 nm from the bottom of the cell are excited [178]. This process allows one to visualize the attachment, assembly, and turnover of the FA.

A migrating cell often experiences rapid re-organization of its FA sites. Understanding the spatial regulation of each site gives a more detailed picture of cell migration. Major advances to the field of optical microscopy now allow the precise visualization of a single fluorescent molecule [179–181]. To supplement these discoveries, efficient algorithms have been developed for single-particle tracking [182]. Super-resolution microscopy that is based on single molecule localizations includes photoactivation localization microscopy (PALM), stochastic optical reconstruction microscopy (STORM), ground state depletion microscopy followed by individual molecular return (GSDIM) and universal point accumulation imaging in the nanoscale topography (uPAINT) [183, 184]. iPALM is a unique form of super-resolution microscopy that combines PALM with simultaneous multi-phase interferometry of photons from individual fluorescent molecules [185]. This technology allows one to image fluorescence-tagged FA proteins with three-dimensional nanoscale resolution [186]. This imaging was used by Kanchanawong, *et al* to determine the three-dimensional localization of many FA proteins including FAK, Paxillin, Talin, Vinculin, and Actin [186]. Super resolution techniques such as iPALM continue to be useful for defining the molecular architecture of FAs.

Some other novel imaging techniques include measuring nanomechanics through atomic force microscopy (AFM) [187]. Time-lapse AFM of live cells permits the observation of the dynamic rearrangements that occur at the cell membrane and the underlying cytoskeleton. AFM allows for the measurement of Young's (*E*) and shear (*G*) modulus [188]. Since mechanical tension sensed by Integrins directs the formation of FAs, AFM provides an excellent opportunity to measure cell traction force. The development of more novel imaging techniques would help our understanding of FA dynamics.

**Figure 5:**
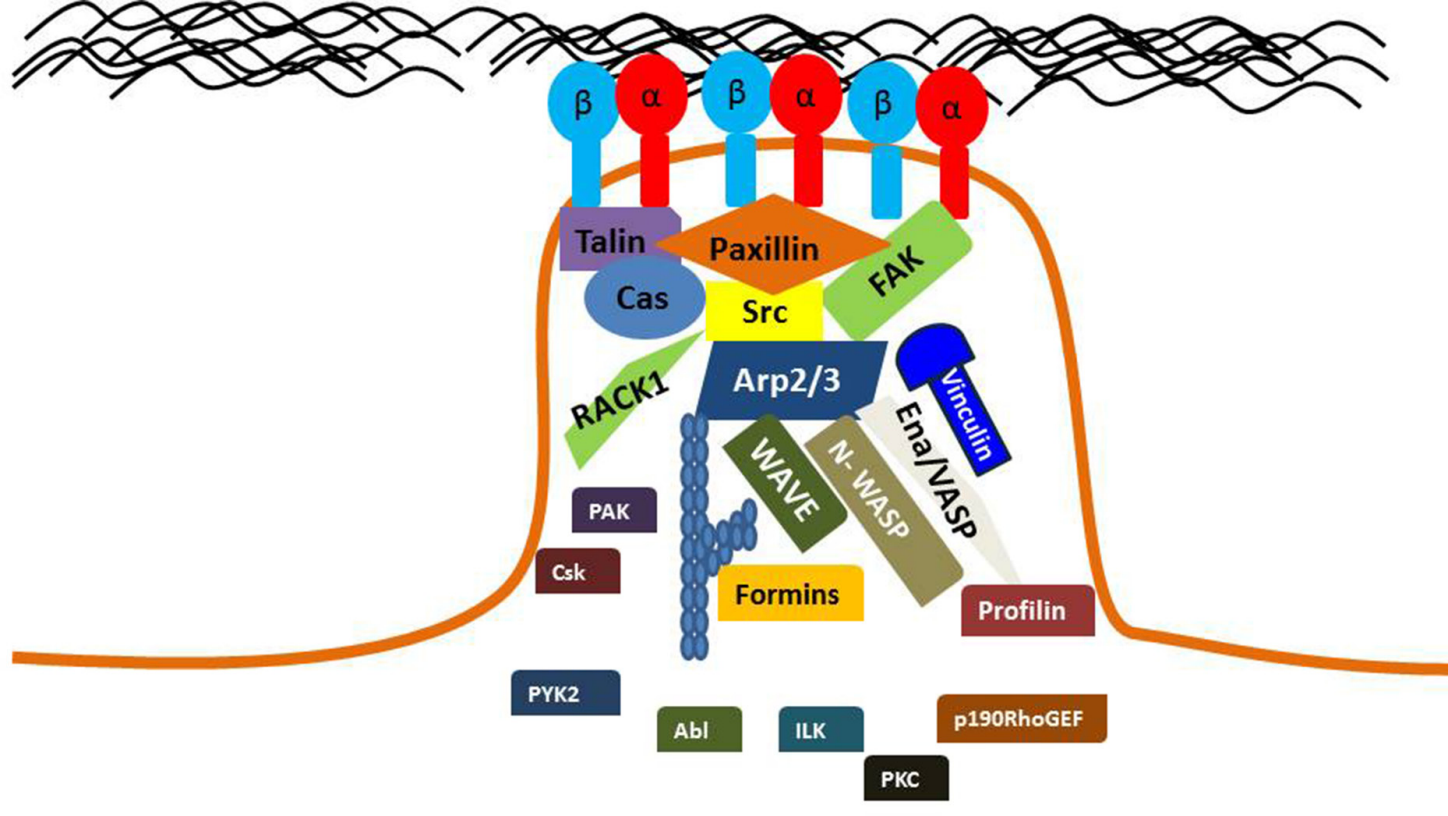
Key proteins are localized to mature FAs Diverse proteins participate in FA signaling, including tyrosine kinases, phosphatases, G-Actin binding proteins, and GTPases. A mature FA will contain activate Integrins that are bound to their ECM ligand. FA signaling pathways regulate the activation of these Integrins.

A cells interaction with its ECM regulates a number of cancer cell processes, including proliferation, migration, survival, and morphology. Specifically, Integrin-mediated adhesion regulates cell signaling, migration and cell contractile force. Alterations in the adhesion mechanics of cells may play a role in the development and progression of cancer. A diverse array of proteins participate in FA signaling, including tyrosine kinases, phosphatases, G-Actin binding proteins, and GTPases as shown in Figure 5. Significant progress has been made in identifying key proteins involved in adhesion signaling, as well as linking these incidents with oncogenic events. Although many proteins in the FA-signaling pathway have been discovered, many more undiscovered proteins may contribute to this phenomena. Fully understanding the unique mechanisms of Integrin-mediated cell adhesions in different cancer types could provide new diagnostic or prognostic markers. To facilitate this, development of novel high-resolution microscopy/spectroscopy methods could aid the field in understanding the complex molecular interactions that occur at specific FA points.
